# Bilateral internuclear and internal ophthalmoplegia due to artery of Percheron infarction

**DOI:** 10.1002/ccr3.837

**Published:** 2017-02-01

**Authors:** Pushpa Raj Puri, Astha Sijapati

**Affiliations:** ^1^ Department of NeurologySlagelse HospitalSlagelseDenmark; ^2^ Department of RadiologySlagelse HospitalSlagelseDenmark

**Keywords:** Bilateral mesencephalic infarction, bilateral thalamic infarction, MRI with angiography, Weber syndrome

## Abstract

The artery of Percheron (AOP) infarction always manifests as paramedian bilateral thalamic infarcts and might present as paramedian midbrain infarcts. Despite the limited MRA evaluation, due to small size of the artery, careful evaluation of the patient's history, the clinical presentation with imaging findings can facilitate the proper diagnosis.

## Introduction

Ischemia of the midbrain and thalami, as well as of the temporal and occipital lobes, can result due to the occlusion of the rostral portion of the basilar artery. Basilar artery occlusions have been well recognized as Kubik and Adams 1 first suggested that this condition could be diagnosed during life. The thalami and the midbrain also receive their blood supplies from the anterior (internal carotid arteries) circulation 2. Castagne et al. 3 and Percheron 4 described the variations in the number and distribution of these perforating arteries and of the infarcts that result from occlusion of the posterior cerebral artery or these branches. One rare variation that supplies bilaterally to the paramedian thalamus and midbrain is the artery of the Percheron (AOP), which arises from one of the proximal segments or P2 segment of the posterior cerebral artery (PCA).

We report a unique case that demonstrates extremely rare and profound neuro‐ophthalmological and behavioral disturbances caused by AOP supplying the rostral midbrain and both paramedian thalami with Weber syndrome due to occlusion of the rostral portion of the basilar artery.

## Case Report

A 64‐year‐old woman, with hypertension and 1 year of left cerebellar infarction was admitted due to sudden early morning onset of dizziness, followed by unconsciousness and left‐sided weakness. There was no history of neck manipulation, trauma, or pain.

**Figure 1:**
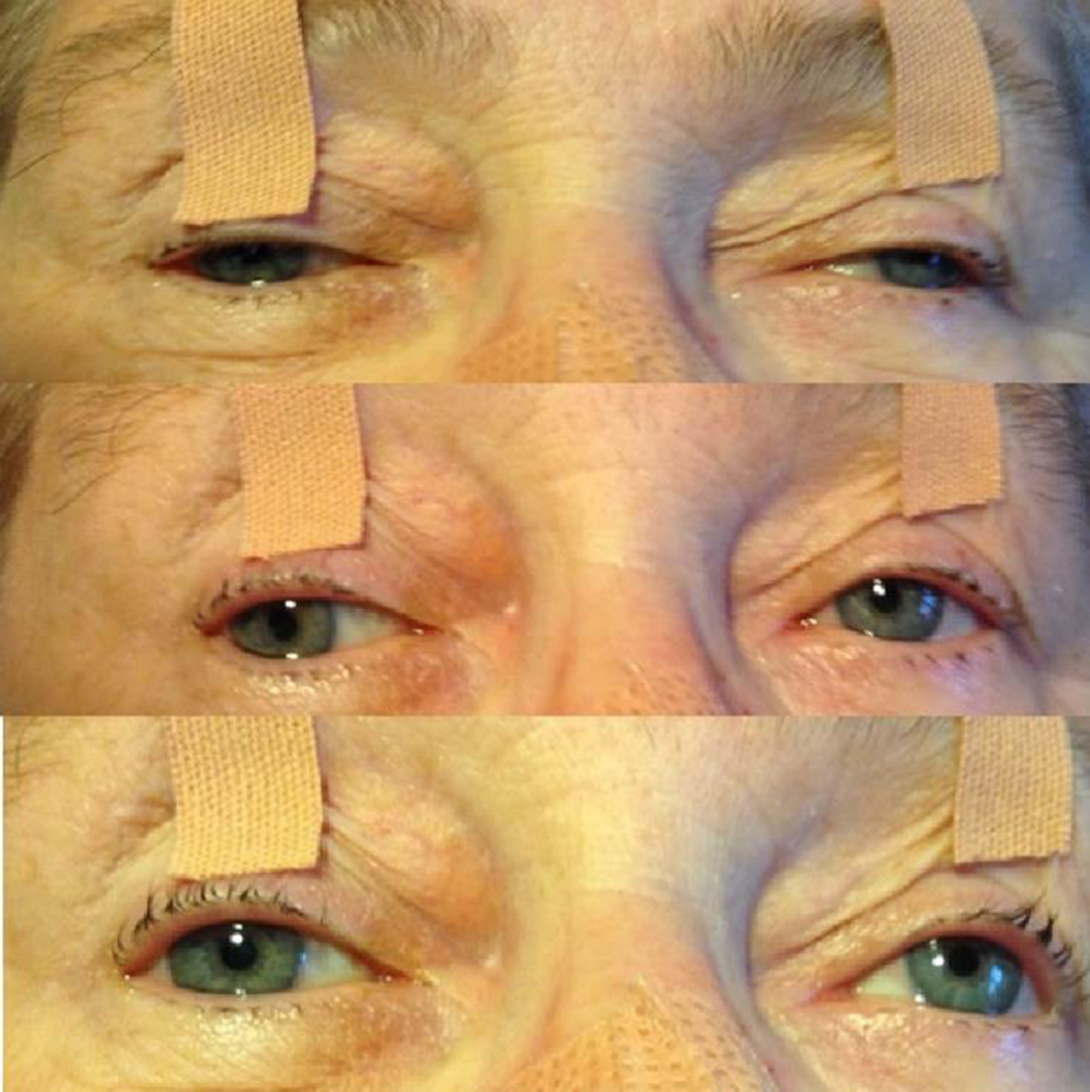
Photograph of the patient's eyes. The eyelids are manually taped because of the bilateral ptosis. The pupils are nonreactive, with an anisocoria (right greater than left).Weak adduction of top: the right eye; middle: left eye; bottom: slight upward gaze palsy of the right eye.

On arrival to the ER, the patient was somnolent and dysarthric with a Glasgow coma score of 8. She reacted well to external stimuli and followed verbal orders. However, in the absence of continuous external stimulation, she fell asleep within a few moments. Cranial nerve examination revealed absence of pupillary reaction to light and anisocoria (Fig. 1), where the right and the left pupil measured 5 and 4 mm, respectively (bilateral internal ophthalmoplegia). She had bilateral ptosis and upward gaze palsy of the right eye (Fig. 1) (bilateral incomplete external ophthalmoplegia), along with a slight left‐sided facial drooping and left‐sided deviation of uvula. She had weak adduction of both eyes and abduction nystagmus of the contralateral eye (bilateral internuclear ophthalmoplegia). She had normal facial sensory response, however decreased sensation in the upper extremities on the left side. Motor function showed left‐sided hemiplegia with grade 1/5 in upper extremities and 2/5 in lower extremities. Deep tendon reflexes on the left side were weak and an upgoing plantar response was found on the same side.

A brain computed tomography (CT) scan performed on the same morning showed normal findings. Electrocardiogram showed sinus tachycardia with AV‐block grade I. Furthermore while in the ER, patient had irregular pulse from 80 to 110. Her situation continued to worsen during the day of admission. The situation was complicated by dysphagia which led to aspiration pneumonia, further worsened by the patients’ refusal to have a nasogastric tube. Later on, percutaneous endoscopic gastrotomy (PEG) was planned, and the patient was given subcutaneous dalteparin, because oral anticoagulants could not be given. She mainly slept during the day and had episodes of confusion and mild agitation during the night.

**Figure 2:**
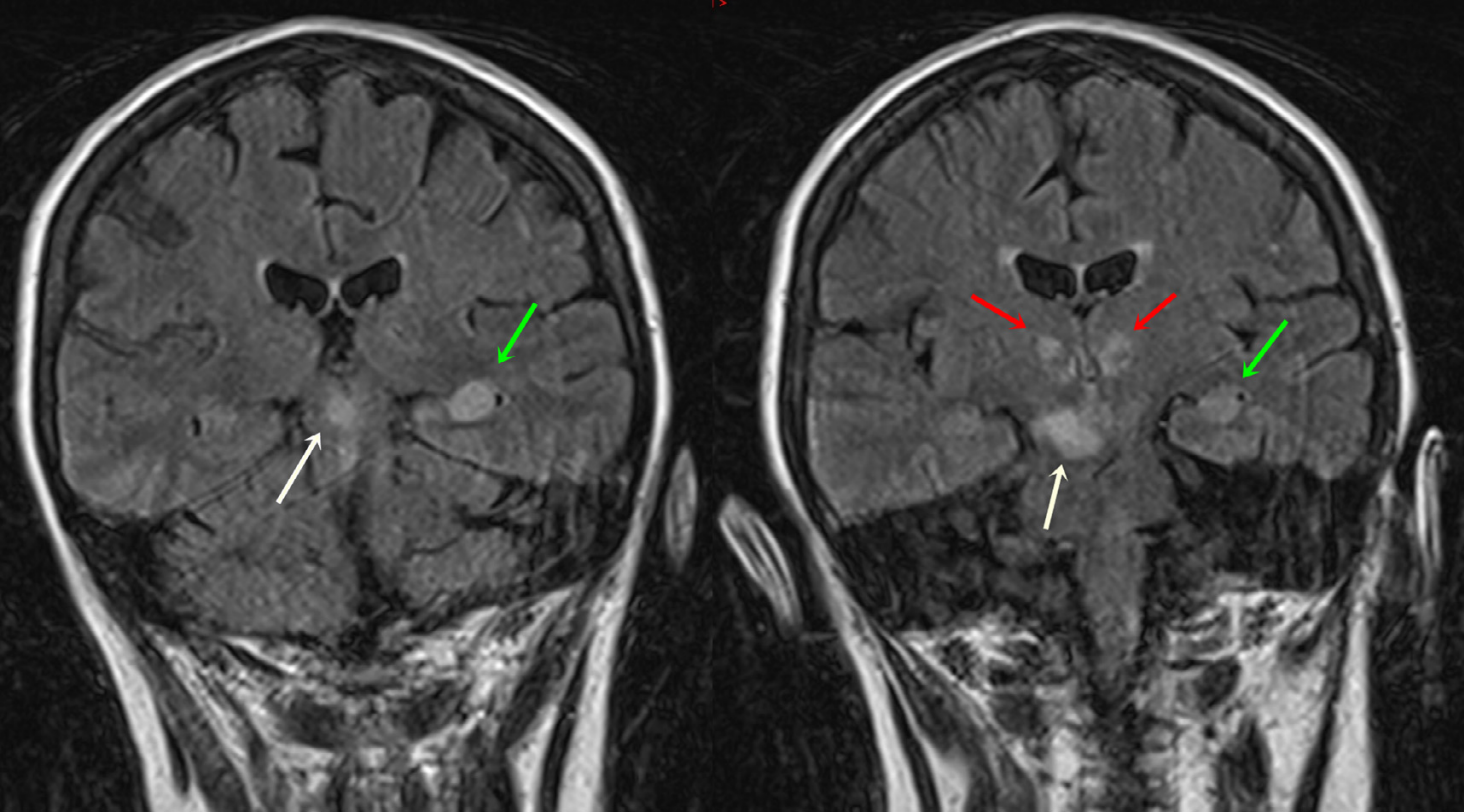
FLAIR images show hyperintense lesion in the central portion of the midbrain (white arrows), bilateral thalami (red arrows), and left hippocampus (green arrows).

**Figure 3:**
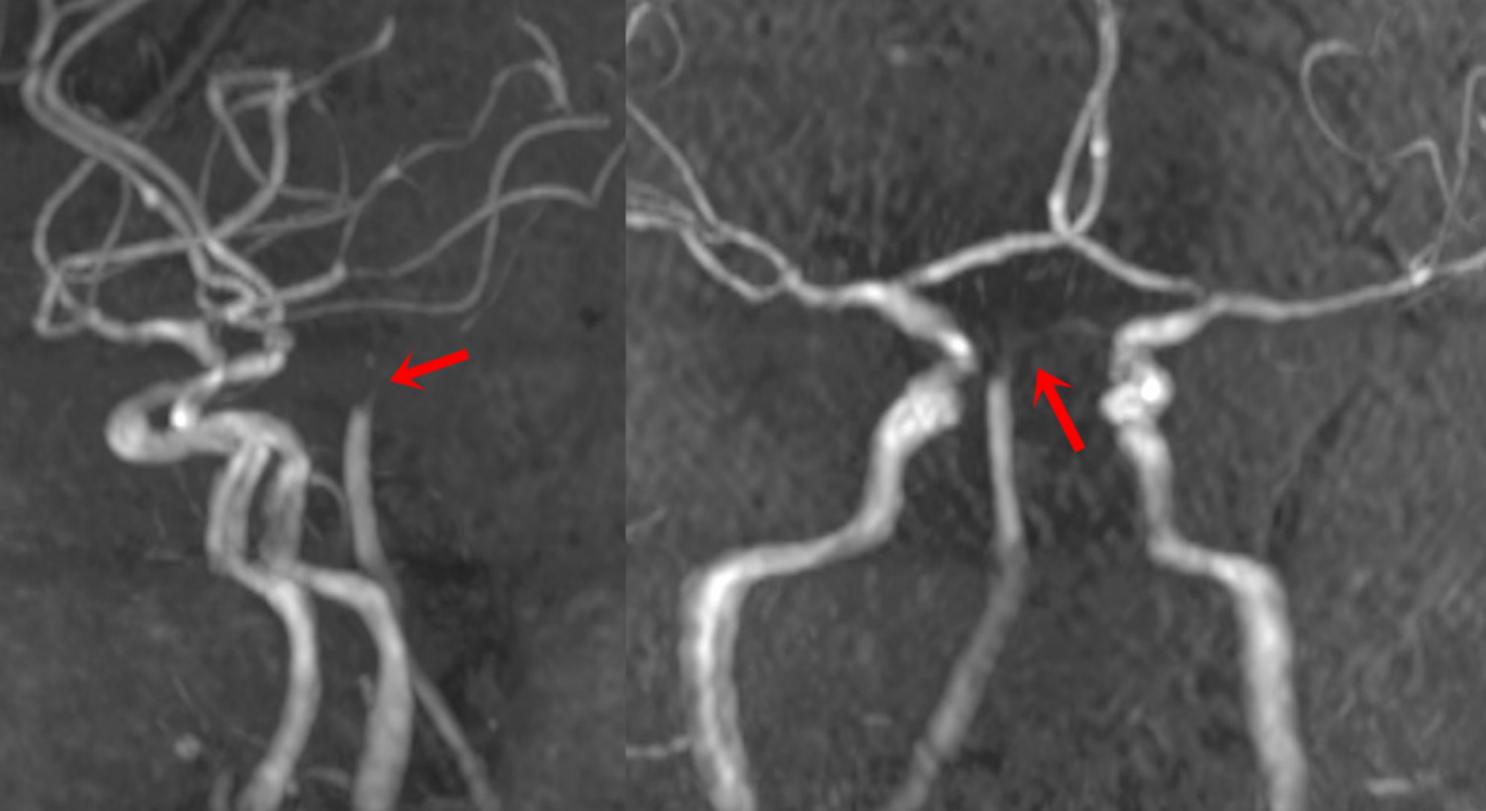
Magnetic resonance angiographic images demonstrating hypoplastic basilar artery ending in superior cerebellar arteries (red arrow).

**Figure 4:**
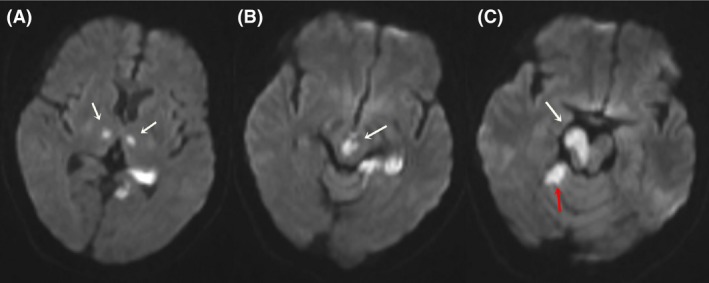
Axial trace diffusion‐weighted images (3500/109[TR/TE], b = 1000 sec/mm^2^) show areas of high signal intensities at (A) bilateral thalamic areas (white arrows), (B) paramedian midbrain (white arrow), and (C) right ventro‐medial part of the upper mesencephalon(white arrow)and right cerebellum (red arrow).

MRI cerebrum with angiography was performed 2 days following admission, which showed brisk infarcts in the rostral central portion of the midbrain, ventral to the aqueduct, affecting the paramedian structures involving the right ventro‐medial midbrain and right cerebellar hemisphere near vermis (Fig. 2). Apart from these findings, there were bilateral paramedian thalamic infarcts involving left hippocampus (Fig. 2). MR angiographic images (Fig. 3) demonstrated low blood flow in the hypoplastic basilar artery, which seemed to end in superior cerebellar arteries. DWI images (Fig. 4) show bilaterally symmetrical high signal intensity in the thalamus, left hippocampus, in the central portion of the upper mesencephalon affecting paramedian structures bilaterally and, the right ventromedial part of mid‐mesencephalon, involving the right cerebellar hemisphere close to the vermis.

Computed tomography angiography of the neck performed on the fourth day of admission showed a slim left vertebral artery with the flow‐restricting stenosis at the caudal part. Echocardiography performed on the same day showed no cardiac source to emboli. On the ninth day, patient showed AF and was treated with digoxin.

The patient showed signs of improvement after 7 days of admission where she was more conscious, less agitated, cooperating well with physiotherapy, and was more awake during daytime. She could slightly lift her eyelids, but showed minimal improvement with her hemiparesis. Later on, a PEG was placed and the patient was discharged on the 25th day to our rehabilitation center for further rehabilitation and physiotherapy.

## Discussion

Occlusion of the rostral basilar artery termed as “top of the basilar syndrome” is known to present a variety of clinical manifestations including visual, oculomotor, and behavioral dysfunctions 5. The majority of our patient's neurological picture can be attributed due to infarction of the paramedian thalami and midbrain as with previously reported similar cases 2. Paramedian midbrain–thalamic infarction results from the occlusion of the midbrain–thalamic perforating arteries, which arise from the proximal PCAs or from a single artery (the artery of Percheron), which in turn arises from one of the PCA. Occlusion of the AOP, as first described by Percheron in 1973, leads to the bilateral paramedian thalamic infarcts with or without midbrain involvement. Castagne et al. 3 stated that, in all cases of this type of arterial pattern or AOP, “the thalamic infarct is always bilateral,” which was also present in our patient. In our case, it is possible that paramedian midbrain infarction with the involvement of the right ventromedial midbrain is caused by occlusion of the median peduncular branches of the mesencephalic arteries from the right PCA 6, although autopsy reports of Lepore et al. 7 have demonstrated such infarction due to the occlusion of the left AOP. So both variants leading to the cause of paramedian midbrain infarction in our case are possible.

The major abnormalities associated with the rostral brainstem infarction involve alertness (disturbances of consciousness, hypersomnia), behavior, memory, pupillary functions, and oculomotor functions, together with bilateral internuclear ophthalmoplegia (INO) as in our case.

Bilateral thalamic infarcts are rare occurrences 8, 9. Patients can present with drowsiness or confusion to hypersomnolence or coma. Our patient was hypersomnolent in the initial days where she had long recurrent episodes of excessive daytime sleepiness, and was agitated during the nighttime, which is one of the well‐known manifestations of bilateral thalamic ischemic stroke 10, 11. Sleep–wake cycle alterations with hypersomnolence can be caused by the involvement of the reticular activating system and the lateral hypothalamus 12. Several differential diagnosis of bilateral thalamic lesion has been discussed previously 13.

Paramedian midbrain infarcts are often associated with nuclear syndrome of the III nerve 14, 15, resulting in bilateral internal and incomplete external ophthalmoplegia as in our case. According to Warnick's scheme 16, the bilateral ophthalmoplegia may result from bilateral pontine and midbrain lesions leading to the third nuclear nerve lesion in which the Edinger–Westphal nucleus is damaged. However, no pontine abnormality was present in our patient. Depending on the location of the lesions, size of the pupil varies, that is, Edinger–Westphal nucleus (large pupils), descending sympathetic fibers (small pupils), or combination of these structures (midrange pupils). The latter is most likely in our case.

Another one of the rare manifestations of vertebro‐basilar ischemia is bilateral internuclear ophthalmoplegia 18, 19, which results from injury to the medial longitudinal fasciculus (MLF) 20 within the dorsomedial pontine or midbrain tegmentum. In our case Figure 4B clearly shows high signal intensity in posterior midbrain in the midline (white arrow) that encircles the periaqueductal gray in the midbrain, where the MLF exists as a pair of white matter tracts. Because of their close physical proximity, bilateral injury is common. The most common causes of INO are multiple sclerosis in the young and brainstem infarction in the adults 20.

If we take the right‐sided oculomotor palsy alone along with the contralateral hemiplegia and slight left‐sided facial droop, then our patient features classical signs of Weber syndrome as described in the literature, which can be well explained by the lesion in the right ventro‐medial midbrain, known to result from the occlusion of the posterior cerebral artery as shown in Figure 4C, which is one of rare manifestations of the midbrain infarcts 17.

Dysphagia in our patient can be assumed to be due to infarction in the right cerebellum, causing lesion to the subset of afferents of vestibular nuclei that project directly to the cerebellum 21. As the dominant left hippocampus primarily mediates verbal learning and memory 22, these are yet to be assessed because our patient up to the present moment did not show any signs of memory loss and had slight dysarthria.

Even though the source of such an embolus was not found in our patient, an atrial fibrillation requiring digoxin certainly raises the possibility of a cardiac mural thrombus. Various major risk factors has been discussed 23, hypertension 17, 23 being the major one, which was present in our patient.

Our case was unusual as the infarcts produced bilateral internal and internuclear ophthalmoplegia in addition to bilateral ptosis. Although there have been reports of complete ophthalmoplegia previously, to the best of our knowledge, the combination of paramedian bilateral thalamic and mesencephalic infarcts with bilateral internal and internuclear ophthalmoplegia in addition to bilateral ptosis and contralateral hemiparesis has not been reported previously.

Even though intra‐arterial thrombolysis and anticoagulation have been proven to be good treatment options for the occlusion of the rostral portion of basilar artery and AOP infarctions 24, it requires intervention within the first few hours after the onset of stroke. As suggested by Jauch et al., due to the hemorrhagic effect, thrombolytic therapy administered many hours or days after the onset of stroke can be dangerous 25. We did not follow the procedure of intra‐arterial thrombolytic therapy, as our patient arrived at the emergency room with the onset of decreased consciousness beginning five hours prior to admission.

Comparatively with the lesions of the cerebral cortex or other subcortical structures, prognosis following thalamic infarction is regarded good. This generally applies to low mortality and probably good recovery from motor deficit 11. In our patient, the level of consciousness gradually improved over the following days; she could open the eyes slightly (left>right) and was more alert during daytime despite fluctuating hypersomnia. Her ophthalmoplegia and pupils remained unchanged up to the present moment.

## Conclusion

Finally, this report demonstrates profound neuro‐ophthalmological and behavioral disturbances caused by the rare but well‐described AOP supplying the rostral midbrain and both paramedian thalami with occlusion of the rostral portion of the basilar artery. Suspicion of occlusion of AOP is raised due to involvement of the paramedian thalamic territories. Performing conventional angiography may not be indicated, because lack of visualization of the artery does not exclude its presence because it is occluded. Rarely, the obstruction of the artery of Percheron can be seen by angiography. Despite the limited MRA evaluation due to small size of the artery, careful evaluation of the patient's history, with the clinical presentation together with imaging findings, can facilitate the proper diagnosis.

## Authorship

PRP: is the corresponding author. AS: has contributed by providing the images with brief explanations and helping with the discussions.

## Conflict of Interest

None declared.
